# PharmRL: pharmacophore elucidation with deep geometric reinforcement learning

**DOI:** 10.1186/s12915-024-02096-5

**Published:** 2024-12-31

**Authors:** Rishal Aggarwal, David R. Koes

**Affiliations:** 1 Joint PhD Program in Computational Biology, Carnegie Mellon University-University of Pittsburgh, Pittsburgh, PA USA; 2 Computational & Systems Biology, University of Pittsburgh, Pittsburgh, PA USA

**Keywords:** Pharmacophores, Virtual screening, Protein-ligand interactions, Machine learning

## Abstract

**Background:**

Molecular interactions between proteins and their ligands are important for drug design. A pharmacophore consists of favorable molecular interactions in a protein binding site and can be utilized for virtual screening. Pharmacophores are easiest to identify from co-crystal structures of a bound protein-ligand complex. However, designing a pharmacophore in the absence of a ligand is a much harder task.

**Results:**

In this work, we develop a deep learning method that can identify pharmacophores in the absence of a ligand. Specifically, we train a CNN model to identify potential favorable interactions in the binding site, and develop a deep geometric Q-learning algorithm that attempts to select an optimal subset of these interaction points to form a pharmacophore. With this algorithm, we show better prospective virtual screening performance, in terms of F1 scores, on the DUD-E dataset than random selection of ligand-identified features from co-crystal structures. We also conduct experiments on the LIT-PCBA dataset and show that it provides efficient solutions for identifying active molecules. Finally, we test our method by screening the COVID moonshot dataset and show that it would be effective in identifying prospective lead molecules even in the absence of fragment screening experiments.

**Conclusions:**

PharmRL addresses the need for automated methods in pharmacophore design, particularly in cases where a cognate ligand is unavailable. Experimental results demonstrate that PharmRL generates functional pharmacophores. Additionally, we provide a Google Colab notebook to facilitate the use of this method.

**Supplementary Information:**

The online version contains supplementary material available at 10.1186/s12915-024-02096-5.

## Background

**Fig. 1:**
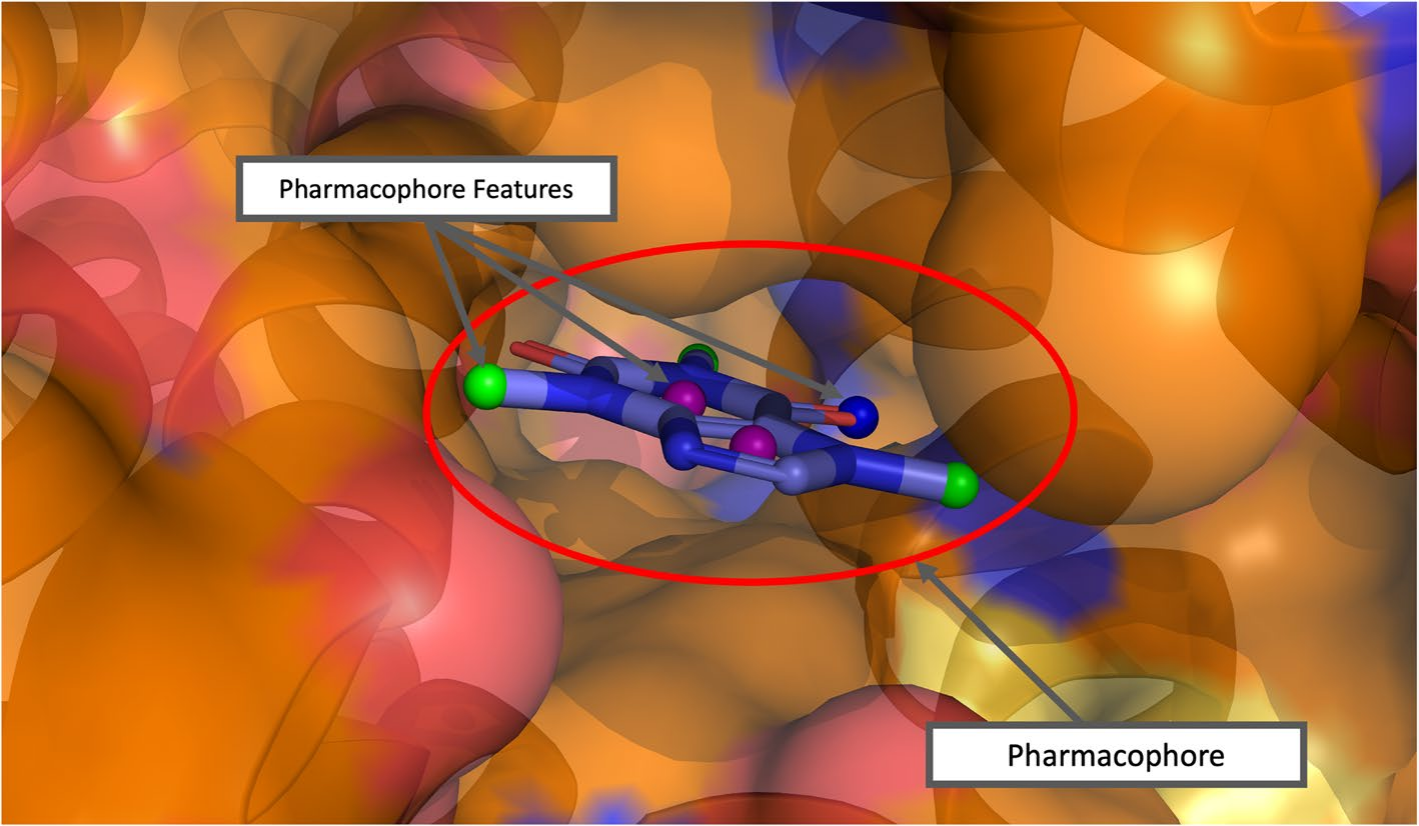
Pharmacophore model with several pharmacophore features that matches the caffeine molecule (caffeine molecule included for illustration). The colors of the feature points are as follows: aromatic—purple, hydrogen acceptor—blue, hydrophobic—green

An essential part of computer-aided drug design is elucidating important molecular interactions between proteins and their ligands. One way to describe these molecular interactions is by depicting a 3D arrangement of protein-ligand interaction features known as a pharmacophore. A pharmacophore is a set of interaction features, also known as pharmacophore features, that describe the favorable interactions between a protein binding site and a ligand. It can be used for screening large libraries through efficient pattern matching algorithms implemented by open source softwares such as Pharmit [1, 2]. An example pharmacophore is shown for the caffeine molecule in Fig. 1.

Pharmacophores are also useful in more situations than just virtual screening. Several machine learning methods take advantage of pharmacophores obtained from co-crystal structures to enhance protein-ligand scoring functions [3, 4]. Furthermore, there have also been a few de novo molecular generation methods that utilize pharmacophores as conditioning and guidance so that generated molecules have desired pharmacophore features [5–8].

Developing a useful pharmacophore typically requires a co-crystal structure of a protein and its cognate ligand. This is because bound structures provide enriched features that can be considered ground truth favorable interactions for protein-ligand binding. This requirement can be a significant obstacle in many real-world drug discovery projects, where bound co-crystal structures are often unavailable.

Traditional methods for generating pharmacophore features in the absence of a ligand typically involve introducing molecular fragment probes into the binding site to identify areas with high affinity [9–11]. Another strategy involves molecular dynamics simulations of the target protein in a simulation environment containing probe molecules with varying chemical properties. This helps pinpoint regions where these probes occur frequently [12]. Subsequently, experts manually select and combine a subset of these interaction features to construct a concise pharmacophore [13, 14]. FRESCO [15] follows a novel approach that avoids filtering features. They use the fit of molecules on distributions of pharmacophore feature distances to rank molecules.

Once interaction features are identified, they need to be ranked and grouped together to form a pharmacophore. Several methods exist for ranking interaction features at binding sites. These methods involve the calculation of interaction energies at feature points [16], while others focus on identifying key pocket atoms for binding [17] and prioritize interaction features in close proximity to these atoms. Recently, a method known as Apo2ph4 [18] was developed for automating the selection process of a subset of pharmacophore features. Apo2ph4 evaluates each feature point by considering both the proximity of other similar features and the interaction energies associated with that point. The resulting pharmacophore is then composed of features whose scores exceed a predetermined threshold. Finally, in certain limited cases, homology models may also be used to elucidate pharmacophores [19, 20]. However, these approaches have notable limitations.

Firstly, the pharmacophore features obtained through these methods are influenced by the biases inherent to the docking and simulation protocols employed. Secondly, the final step of constructing the pharmacophore heavily relies on human insight. Furthermore, methods that try to filter out pharmacophore features evaluate each feature in isolation rather than considering its contribution to a fully-formed pharmacophore. These factors collectively underscore the need for more experimental, data-driven approaches to generate pharmacophore features. Additionally, there is a need for the development of automated tools for pharmacophore modeling that can still benefit from expert guidance.

**Fig. 2:**
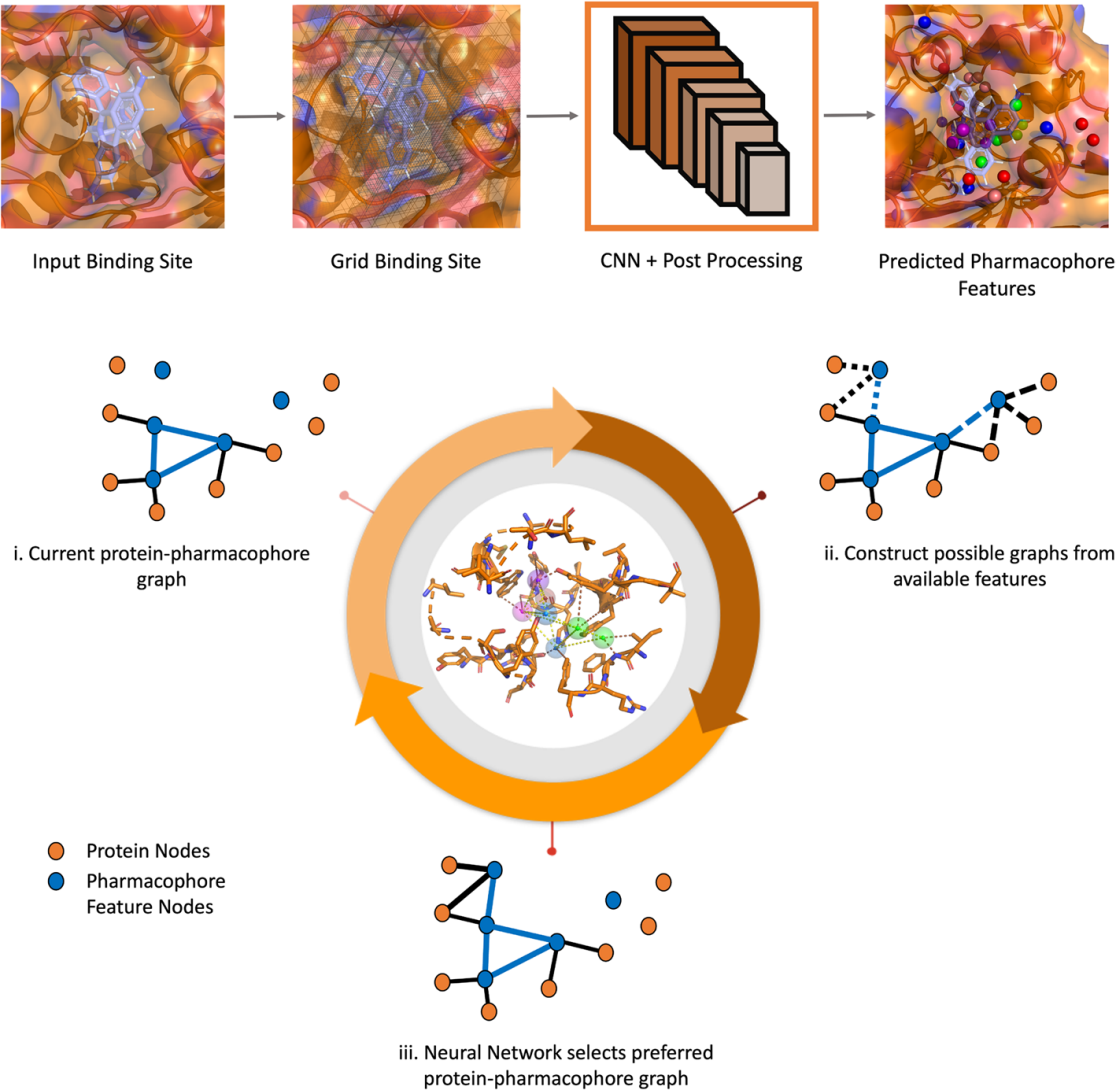
Pharmacophore prediction pipeline. CNN is used to predict pharmacophore features from gridded binding site (top). Protein—pharmacophore graph is built by sequentially adding feature and protein nodes to it using RL framework (bottom)

To address this requirement, we developed a CNN model and a deep geometric Q-learning algorithm to identify interactions features and elucidate pharmacophores. The method is demonstrated to strong performance in retrospective virtual screening experiments across several datasets such as Dataset of Useful Decoys - Enhanced (DUD-E) [21], LIT-PCBA [22], and COVID Moonshot [23].

## Methods

In this method, we trained a convolutional neural network (CNN) model to identify favorable points of interactions (pharmacophore features) on the binding site and developed a deep geometric Q-learning algorithm that attempts to select an optimal subset of these interaction points to form a pharmacophore. The CNN model is trained on pharmacophore features derived from protein-ligand co-crystal structures and is iteratively fine tuned with adversarial examples to ensure predicted points of interaction are physically plausible and close to relevant functional groups on the protein. The reinforcement learning algorithm employs an SE(3)-equivariant neural network [24] as the *Q*-value function. This network progressively constructs a protein-pharmacophore graph. It does so by either choosing to incorporate an available pharmacophore feature into the graph or determining that the current graph is already optimal. The pipeline for the method is shown in Fig. 2. Importantly, this framework still has the ability to accommodate expert guidance in selecting and adding features while automating a significant portion of the traditional pharmacophore elucidation process.

### Pharmacophore definition

A pharmacophore is defined as a set of points $\{V_f\}$ that propose positions of interactions between the give protein binding site and a potential ligand. More specifically each point in a pharmacophore has a 3D coordinate $X_f \in \mathbb {R}^{3}$ and feature class $Z_f$. The feature class is defined to be any of the following: $\{$Hydrogen Acceptor, Hydrogen Donor, Hydrophobic, Aromatic, Negative Ion and Positive Ion$\}$. Pharmacophore search software such as Pharmit [1] can be used to retrieve molecules that can satisfy the feature and position constraints specified by a given pharmacophore. In this work, pharmacophores are developed in two major steps. First, potential points of interactions on a binding site are identified using a convolution neural network (CNN). A subset of these identified points are then selected with a reinforcement learning model to form a pharmacophore. More details follow in subsequent sections.

### Molecular conformation generation and pharmacophore screening

Molecule conformers for pharmacophore screening are generated using RDKit [25] for the DUD-E and Covid Moonshot datasets, with 25 energy minimized conformers produced per molecule. The LIT-PCBA dataset, however, is prohibitively large for conformation generation. Therefore, we submit the list of molecules from this dataset directly to the Pharmit server [26]. This approach saves on compute as Pharmit’s database already contains conformers for most of these molecules due to significant overlap with other datasets hosted on the server. By default, Pharmit stores 20 conformers per molecule. Pharmit is also used to screen pharmacophores on these conformers. The software retrieves and aligns conformers that match the spacial restraints specified by the pharmacophore, with a tolerance radius of 1 Å for all of its features. We also remove conformers that overlap with the protein (receptor exclusion in pharmit) from the screening results. We ensure that only 1 conformer per molecule is returned by the software to calculate our performance metrics.

### CNN training

The CNN model is trained to predict whether a given point on the binding site is a plausible point of interaction. Specifically, the CNN predicts if any of the six feature classes are present at the given point. It is trained in a multilabel classification manner so that it can predict the presence of multiple classes at the evaluated point. This approach accounts for overlap between different classes. For instance, certain aromatic groups can be viewed as hydrophobic, and similarly, some hydrogen acceptor groups may also be regarded as negative ion functional groups with the ability to form salt bridges.

**Fig. 3:**
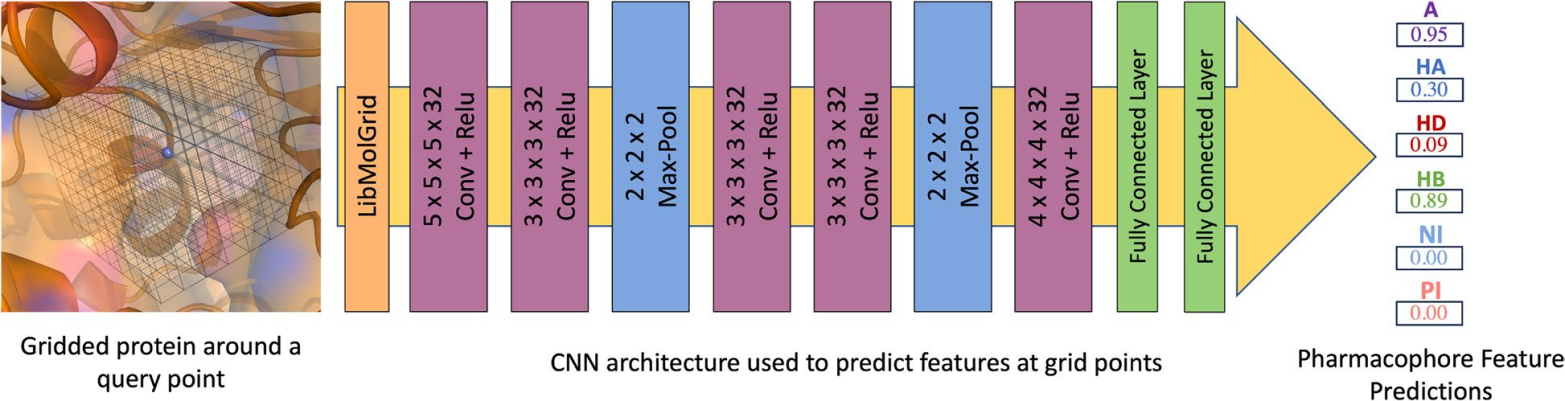
CNN architecture for predicting pharmacophore feature points. The CNN takes the local grid around the query point as input and provides confidence scores on the presence of the 6 classes at that point

The CNN takes as input, a voxelized representation of the protein structure located in a cubic volume of edge 9.5 Å, at a resolution of 0.5 Å, centered at the point. The libmolgrid [27] python library with its default atom types is used for voxelizing the protein structure. The model is trained for 256 epochs, with a batch size of 256, using the adam optimizer at a learning rate of 1e−5. The model checkpoint with the best metrics on the test set is saved. The CNN architecture is provided in Fig. 3.

The model is initially trained on pharmacophore features extracted from the PDBBind V. 2019 dataset [28]. For each structure we use pharmacophore feature interaction points identified by Pharmit as our training samples. The command line used for to extract features for each crystal structure is pharmit pharma -receptor receptor.pdb -in ligand.mol2 -out pharmit.json. The extracted dataset is split into three cross-validation folds with data points (pharmacophore features) from similar ligands being in the same fold. Ligand similarity is determined by Tanimoto similarity over RDKit [25] fingerprints. Two ligands with a Tanimoto similarity greater than 0.9 are considered to be similar and are clustered together into the same fold. In total we have 157,252 data points with approximately 104,835 data points in the training sets and 52,417 data points in the test sets. We train separate models for each fold and use the best performing model for inference.

**Table 1:** Pharmacophore interaction distance thresholds

Pharmacophore feature	Protein feature	Min distance threshold	Max distance threshold
Aromatic	Aromatic	1.5	7
Hydrogen acceptor	Hydrogen donor	1	4
Hydrogen donor	Hydrogen acceptor	1	4
Hydrophobic	Hydrophobic	1.5	5
Negative ion	Positive ion	1.5	5
Positive ion	Negative ion	1.5	5

To enhance the robustness of pharmacophore feature predictions, the CNN undergoes retraining with adversarial samples. Adversarial samples are generated through a two-step process. Firstly, the protein binding sites are discretized at a resolution of 0.5 Å, and the CNN is evaluated at each grid point. Predictions that are too close to protein atoms are labeled as negative. Additionally, predictions where complementary functional groups of interest on the protein are too distant are collected as adversarial samples. For instance, hydrogen acceptor predictions beyond 4 Å from any hydrogen donor functional group on the protein are considered negatives. Thresholds for pharmacophore features and their complementary functional groups are outlined in Table 1. Complimentary functional groups on the protein are found using the same SMART strings as those defined in Pharmit. The adversarial samples are then added as negative data points to the training set to retrain the model.

### From CNN predictions to pharmacophore features

Pharmacophore features are individual interaction points found in the binding site. One key assumption is that these features should be in proximity to complementary interaction feature groups on the protein. These features are inferred through a multi-step process.

**Fig. 4:**
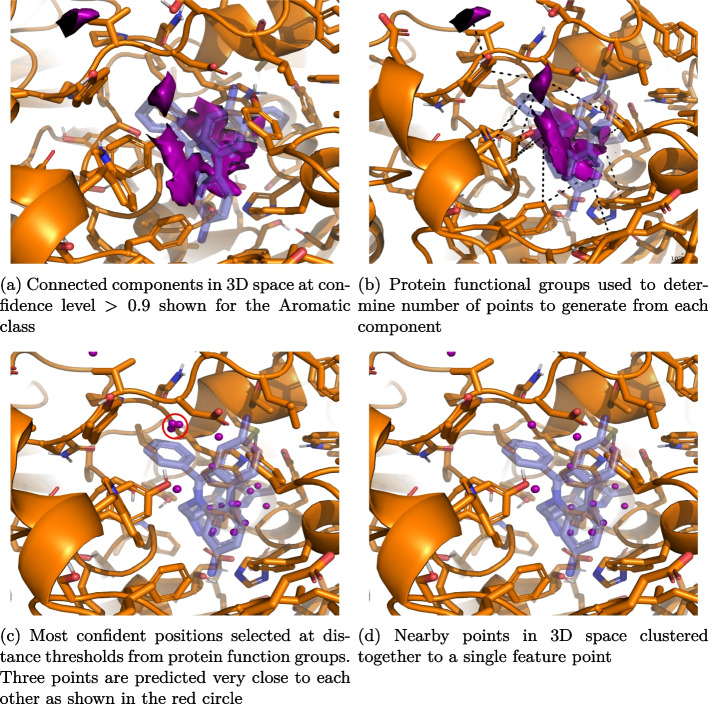
Steps followed to obtain pharmacophore feature points from a CNN predictions on a binding site

The pharmacophore generation process can be viewed in Fig. 4. As before, the binding site is first gridded at a resolution of 0.5 Å and the CNN is evaluated at each grid point. This results in a dense grid of feature confidences. Once this is done we need to determine the number of feature points that have to be extracted from each connected component. We use the complimentary functional groups on the protein that are close to the connected components (Fig. 4b) to determine the number of feature points by taking the top predicted point (Fig. 4c) within a distance threshold (refer to Table 1 for the thresholds) to it.

Finally, feature points are refined by grouping predictions that are near each other through agglomerative clustering. A distance threshold of 1.5 Å is used as criteria for merging clusters and the centroid of each cluster is taken as the predicted pharmacophore feature (Fig. 4d).

### Formation of pharmacophores from features

A subset of the candidate pharmacophore features are selected to form a full pharmacophore. This process is modeled as a reinforcement learning problem. Specifically, the method is a deep Q-learning framework that utilizes a SE(3)-equivariant neural network [24] to model the Q value function. The RL algorithm is trained on the Dataset of Useful Decoys - Enhanced (DUD-E) dataset as it provides an extensive set of actives and decoys for each protein-ligand system in its dataset.

### Why reinforcement learning

Modeling pharmacophores presents a substantial challenge because it involves selecting a concise set of features suitable for virtual screening. Pharmacophores are built by combining specific features, and this combination greatly influences their performance. Notably, adding or removing a single feature can significantly impact the overall performance, making it challenging to assess the individual importance of each feature in isolation. This complexity poses a hurdle for traditional supervised learning approaches, such as the CNN.

However, reinforcement learning (RL) offers a different perspective. RL has the potential to consider the long-term consequences of adding a single feature to a pharmacophore. Consequently, a RL algorithm can sequentially incorporate features into a pharmacophore model while considering the overall value of the fully formed pharmacophore, rather than just the immediate value of each individual feature added along the way.

### Pharmacophore selection as a Markov decision process (MDP)

The generation of the pharmacophore follows an iterative process via the construction of a heterogeneous 3D graph. The graph contains “pharmacophore” nodes ({$V_f$}) representing pharmacophore features and “protein nodes” ({$V_p$}) that contain the protein atoms in proximity of the bespoke pharmacophore features. Each iteration involves adding pharmacophore feature nodes and their associated protein atoms to the graph. The structure of the graph in the next step depends entirely on its current state, making the process akin to a Markov decision process (MDP).

In the context of reinforcement learning, a Markov decision process (MDP) is defined with a set of states $s \in S$ that provide information of the environment, actions $a \in A$ that help in moving from the current state to the next state , and a reward function $R(s,a) \rightarrow \mathbb {R}$ that provides a reward value for state-action pair.

**Fig. 5:**
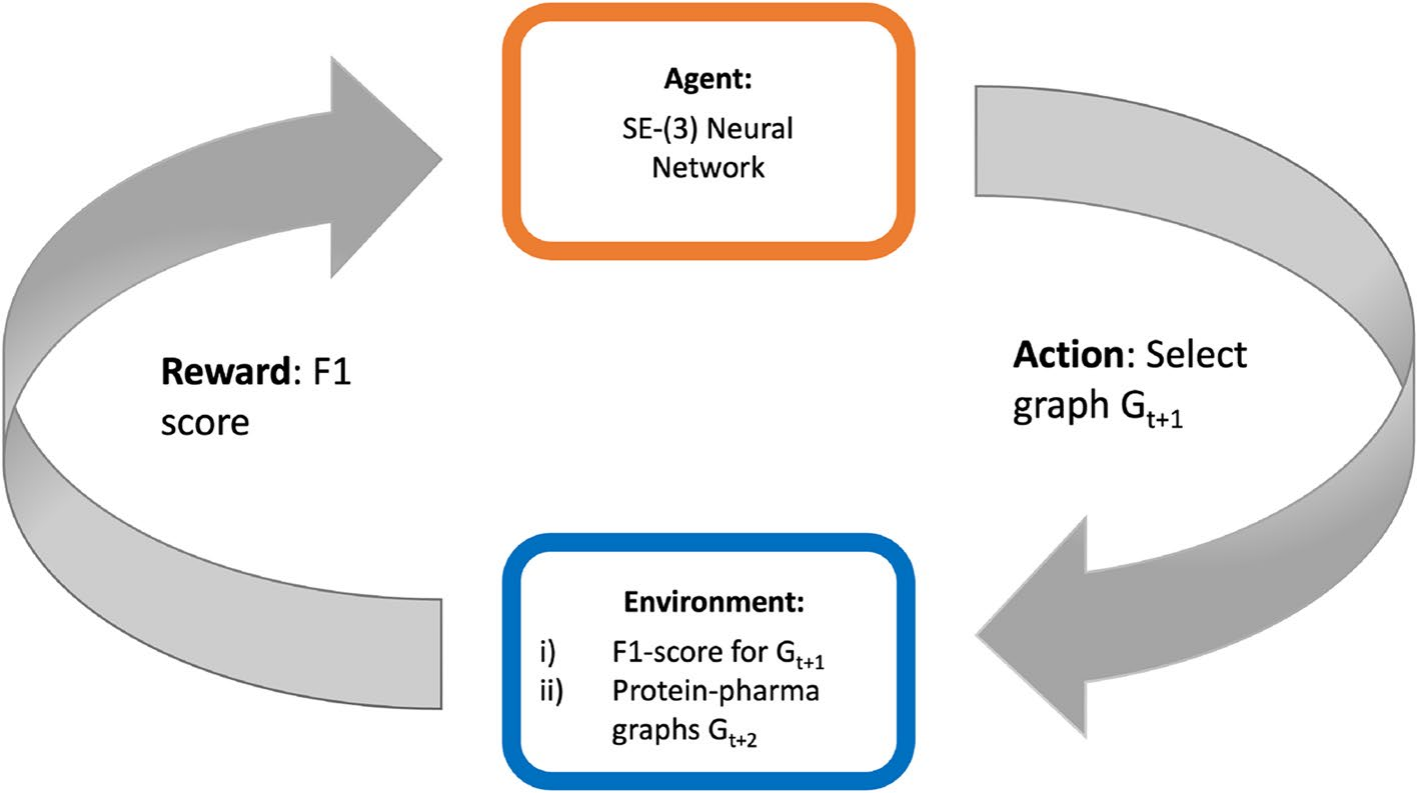
MDP process used for iterative construction of the protein-pharmacophore graph. At each time-point *t*, the action is to chose the next graph ($G_{t+1}$). The environment takes this and provides a *F*1 score for that pharmacophore, along with possible graphs to choose from ($\{G_{t+2}\}$) for the next iteration

Here, at a given time-point (*t*) in the iterative process, we define a state ($s_t$) as a heterogeneous protein-pharmacophore graph denoted as $G_t(V_f,V_p,E_{f,f},E_{f,p})$, consisting of pharmacophore feature nodes ($V_f$), protein nodes ($V_p$), and edges ($E_{f,f}$ and $E_{f,p}$) connecting feature nodes to features and protein nodes. The edges are formed based on predefined distance thresholds $\delta _{f,f}$ and $\delta _{f,p}$.

This definition leads to a set of possible states $\{s_{t+1}\}$ that can be reached at time-point $t+1$ by considering the addition of a feature not present in the current graph but within a distance of $\delta _{f,f}$ from any feature node in the graph. This results in a set of proposed graphs denoted as $\{G_{t+1}\}$. The current graph $G_t$ is also added to this set, forming a superset $\{s_{t+1}\} = \{ \{G_{t+1}\}, G_t \}$. The action ($a_t$) then involves selecting one of the graphs from this proposal set as the next state. If the current graph is selected, the process terminates.

The reward for each step $r_t=R(s_t,a_t)=R(G_{t+1})$ is calculated based on the *F*1 score obtained by running the pharmacophore, obtained as a combination of the features nodes in the graph $G_{t+1}$, on a dataset containing actives and decoys. Pharmit, the tool used, requires a pharmacophore with at least 3 nodes to screen molecules. Therefore, until the current graph has at least 3 nodes, we assign a reward of 0 and do not include the current graph in the proposal set. A schematic representation of the MDP process at time-point *t* is given at Fig. 5.

### Deep Q-learning

The objective of reinforcement learning is to learn a policy $\pi ^*:S \rightarrow A$ that maximizes the cumulative (discounted) reward you obtain from a MDP. In Q-learning, the function *Q*(*s*, *a*) is trained to predict future rewards given an action on a state. In this context, for a policy $\pi$ the *Q*-value of a state action pair is given by:

1 $$\begin{aligned} Q^\pi (s_t,a_t) = Q^\pi (G_t,a_t) = Q^\pi (G_{t+1}) = \mathbb {E} \left[ \sum \limits ^T_{i=t} \gamma ^{i-t}*r_i\right] \end{aligned}$$

where $\gamma$ is a predetermined reward discount factor. The discount factor implicitly weighs the importance of the immediate reward with respect to the cumulative reward. The optimal policy defined at a state then is $\pi ^*(s) = {argmax}_a Q^{\pi ^*}(s,a)$. For this problem this equation translates to:

2 $$\begin{aligned} \pi ^*(G_t) = argmax_{G_{t+1}}Q^{\pi ^*}(G_{t+1}) \end{aligned}$$

**Fig. 6:**
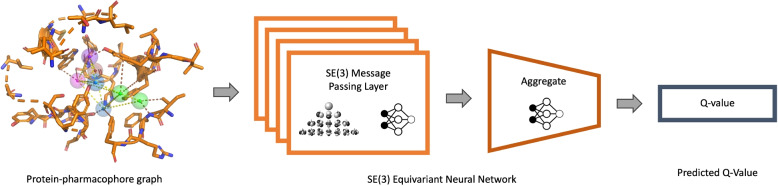
The SE(3)-equivariant neural network takes a protein-pharmacophore graph as input and predicts the *Q*-value

A SE(3)-equivariant neural network is used to parameterize the Q function (Fig. 6). The neural network is trained to minimize the objective $l(\theta ) = \mathbb {E}\left[ y_t - Q(G_t;\theta )\right]$ where $\theta$ is the parameter set of the neural network and $y_t$ is given by:

3 $$\begin{aligned} y_t = r_t + \gamma * max_{G_{t+2}}Q(G_{t+2};\theta ) \end{aligned}$$

### Graph featurization

We construct heterogenous graphs $G(V_f,V_p,E_{f,f},E_{f,p})$, consisting of pharmacophore feature nodes ($V_f$), protein nodes ($V_p$), and edges ($E_{f,f}$ and $E_{f,p}$) connecting feature nodes to features and protein nodes. Since we model a 3D graph, each node has a 3D coordinate in addition to node features. Therefore, we can construct our edges $E_{f,f}$ and $E_{f,p}$ using appropriate distance thresholds ($\delta _{f,f}$) and ($\delta _{f,p}$). The thresholds themselves were decided through hyperparameter sweeps. The node features for the protein nodes are one-hot encodings of the atom types defined by the libmolgrid library. The atom types are listed in Table 2. The node features for the interaction feature nodes are the output of the final hidden layer of the CNN. We use the output from the CNN as it is essentially an embedding of the local information around that point. The edge features provided to the model are a radial Gaussian basis embedding of the edge distance.

**Table 2:** Atom types used to featurize protein nodes

Atom type name	Atom type number
AliphaticCarbonXSHydrophobe	1
AliphaticCarbonXSNonHydrophobe	2
AromaticCarbonXSHydrophobe	3
AromaticCarbonXSNonHydrophobe	4
Bromine Iodine Chlorine Fluorine	5
Nitrogen NitrogenXSAcceptor	6
NitrogenXSDonor NitrogenXSDonorAcceptor	7
Oxygen OxygenXSAcceptor	8
OxygenXSDonorAcceptor OxygenXSDonor	9
Sulfur SulfurAcceptor	10
Phosphorus	11
Calcium	12
Zinc	13
GenericMetal Boron Manganese Magnesium Iron	14

### Q-function neural network

We train an SE(3)-equivariant graph neural network as our Q-function. The neural network consists of separate embedding layers for the different node and edge types, *k* message passing layers, a global mean pooling aggregation layer and a final fully connected layer that predicts the *Q*-value. The input graph has two types of edges: feature node–protein node and feature node–feature node. To model this heterogeneity we have separate message passing weights for the two edge types.

The message passing layers in our model utilize SE(3)NN convolution layers, implemented through the e3nn Python package [29]. These convolution layers are based on a spherical harmonic basis with varying orders represented by “*l*” and operate on scalar features ($l=0$), vector features ($l=1$), and higher-order features ($l>1$). In our implementation, we do not exceed $l=2$. Additionally, for the convolution, we model our edge features by a concatenation of the scalar features of the nodes involved and the edge embedding.

The SE(3)NN commences with basic scalar node features and progressively generates higher-order features with both odd and even parity as the network deepens, accomplished through tensor product convolutions. To determine the network’s width, we define two parameters: *ns* determines the number of scalar features produced by each layer, while *nv* dictates the number of higher-order features of each type ($l=1$, $l=2$) for both odd and even parity. We use $ns = 32$ and $nv = 8$ for our neural network.

### Training details

The DUD-E dataset is split into training and test sets, with the test set being the diverse subset of the DUD-E dataset. This subset contains 8 proteins that are representative of all the proteins in the dataset. The neural network operates on a protein-pharmacophore graph as input. Protein nodes are represented by atom types, while pharmacophore nodes take as features the output of the final hidden layer of the CNN. The final hidden layer of the CNN can be interpreted as an embedding of the local environment around the feature point. It provides a latent vector of size 32. Initially, the model is trained using pharmacophore features extracted from protein-ligand co-crystal structure and is then fine-tuned using pharmacophore features obtained from CNN predictions. Since the ligand features are obtained from crystal structures, they also have directional information about aromatic/hydrogen bonding interations between the ligand and the protein which are used while evaluating generated pharmacophores. A hyperparameter sweep was also conducted while training on pharmacophore features extracted from the cognate ligand. The model that provides the best mean *F*1 score on ligand extracted features is used to train an ensemble of 5 models on CNN-predicted features.

The training algorithm goes through the protein-ligand systems in the training set, generating training samples through episodes simulated using an $\epsilon$-greedy policy. $\epsilon$-greedy balances exploration and exploitation by setting a probability $\epsilon$ by which a random action is taken as compared to taking the action decided by the neural network. While training the epsilon decays exponentially according to the equation $\epsilon _t=\epsilon _T + (\epsilon _o-\epsilon _T)*e^{(-t/\alpha )}$, where $\epsilon _o$ and $\epsilon _T$ are initial and final epsilons, and $\alpha$ is a predetermined decay rate parameter. Using this, the initial iterations of RL training is focused on exploring as many graphs as possible. Later iterations are focused on optimizing the learnt policy based on the graphs sampled by the neural network as the neural network has better graph proposals. While training on ligand based features, an $\epsilon _o$ value close to 0.9 is used, but when fine-tuning on CNN features $\epsilon _o=0.5$ is used as lesser amount of exploration is required at this stage.

To simulate an episode, we begin by randomly selecting a protein-ligand system from the dataset. Initially, we set up an empty protein-pharmacophore graph as the starting state. In the first step of the simulation, the policy is allowed to select any pharmacophore feature, along with its corresponding protein atoms, to add to the graph. Subsequent steps only permit the addition of a feature node (and its associated protein atoms) if they are within a distance of $\delta _{f,f}$ from the feature nodes already present in the graph. This criterion is used to generate a set of proposed graphs for the next step. Additionally, if the current graph contains at least 3 feature nodes, it is included in this set.

At each step, the policy selects a graph from this proposal set, and the associated reward for that action is collected. This process continues iteratively until either the same graph is selected again or the maximum number of steps (*T*) allowed in an episode is reached. While training on ligand features we set $T = 10$ and on CNN features we set $T=5$

We maintain a replay memory *M* of capacity *N* that stores the latest training samples generated from the simulations. In addition we use a separate target neural network with fixed parameters that provides the target for training the Q function neural network. This stabilizes training of the neural network. Every *C* episodes, the parameters of the target network are updated as a linear combination of the target and Q function network parameters. The importance given to the target network parameters in the update is defined by another parameter $\tau$.

The full training algorithm is provided in Algorithm 1.

**Figure:**
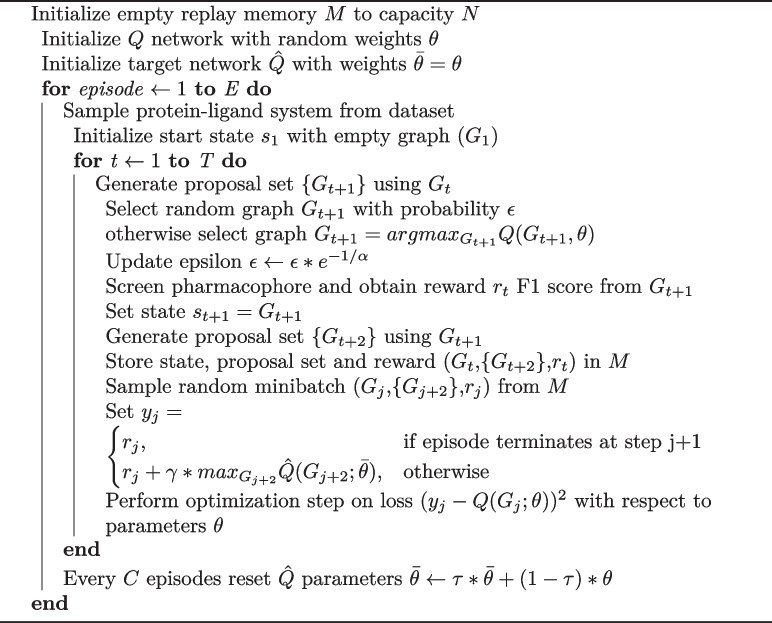
**Algorithm 1** Deep Q-learning algorithm to train Q function network

### Hyperparameter sweep

To identify the optimal combination of parameter values, we perform a Bayesian hyperparameter search during the training process using pharmacophore features from the ligand. We keep track of the mean *F*1 score on the test set for models trained with various parameter combinations, and we select the parameter set that yields the highest *F*1 score.

**Table 3:** Hyperparameter options searched through for RL Q function model

Parameter	Type	Search space	Value
Batch norm	Categorical	True, False	True
Batch size	Integer	Range [16, 64]	50
Epsilon decay$\alpha$	Float	Range [5000, 25,000]	11,967
Epsilon final$\epsilon _T$	Float	Range [0.02, 0.005]	0.017
Epsilon start$\epsilon _o$	Float	Range [1, 0.8]	0.836
Discount factor$\gamma$	Float	Range [1, 0.45]	0.8636
Learning rate	Float	Range [0.001, 0.00001]	0.00012
Memory size *N*	Integer	Range [500, 2000]	1893
Number of message passing layers *k*	Integer	Range [4, 8]	6
Number of episodes *E*	Integer	Range [10,000, 25,000]	16,752
Feature - feature node distance threshold$\delta _{f,f}$	Integer	Range [12, 15]	12
Feature - protein node distance threshold$\delta _{f,p}$	Integer	Range [8, 12]	11
Target update frequency *C*	Integer	Range [1, 10]	2
Target update importance$\tau$	Float	Range [0.5, 1]	0.686

This hyperparameter search is executed through the use of wandb (https://wandb.ai/site). A comprehensive list of hyperparameters and their respective selected values can be found in Table 3.

### Performance metrics

We evaluate the methods presented in this work through several metrics. To calculate these metrics we define the following:

- **True positives (TP)**: # of molecules returned by the pharmacophore that are known to be actives
- **False positives (FP)**: # of molecules returned by the pharmacophore that are known to be decoys
- **True negatives (TN)**: # of molecules not returned by the pharmacophore that are known to be decoys
- **False negatives (FN)**: # of molecules not returned by the pharmacophore that are known to be actives

The metrics we evaluate the methods on are

- **Hit rate**: The hit rate is given by
  4 $$\begin{aligned} HR= \frac{TP + FP}{TP + FP + TN + FN} \end{aligned}$$
- **Precision**: The precision is given by
  5 $$\begin{aligned} P= \frac{TP }{TP + FP} \end{aligned}$$
- **Recall**: The recall is given by
  6 $$\begin{aligned} R= \frac{TP }{TP + FN} \end{aligned}$$
- **F1 score**: The *F*1 score is given by:
  7 $$\begin{aligned} F1= \frac{2*P*R }{P + R} \end{aligned}$$
- **Enrichment factor** The enrichment factor is given by:
  8 $$\begin{aligned} EF = P / \frac{TP+ FN}{TP + FP + TN + FN} \end{aligned}$$
- **Guner-Henry ** The Guner-Henry metric is given by:
  9 $$\begin{aligned} GH = \left[ \frac{TP*(3*(TP+FN)+TP+FP)}{4*(TP+FN)*(TP+FP)}\right] \left[ 1-\frac{FP}{TN+FN}\right] \end{aligned}$$

We place emphasis on the *F*1 score and the enrichment factor for our experiments. The *F*1 score is used as it remains relatively unbiased for an unbalanced dataset. The enrichment factor provides a quantitative comparison on the number of actives in our hits vs the dataset, thus providing how much our screening approach has *enriched* our hits.

## Results

We show that this algorithm has the potential to provide performant solutions in virtual screening experiments on the Dataset of Useful Decoys - Enhanced (DUD-E) [21] and LIT-PCBA [22] datasets. We also test the method on screening the COVID Moonshot dataset [23] and show that it would provide pharmacophores with the ability to identify binding molecules even in the absence of fragment screening experiments.

### CNN models successfully classify pharmacophore feature points

The CNN model is initially trained using features extracted from co-crystal structures. It is worth emphasizing that every data point in this dataset is associated with at least one of the classes, and in such cases, the model accurately predicts the corresponding classes with high precision. The ROC-AUC for each class surpasses 0.95, and detailed class-specific ROC-AUC scores can be found in Table 4. Subsequently, the CNN undergoes retraining using adversarial examples to enhance the robustness of its predictions during inference. Importantly, this retraining has a negligible impact on the model’s classification performance. Furthermore, certain false positives are accounted for by ensuring that generated features are at appropriate distance from the relevant function groups on the protein. The feature prediction algorithm provides an average of 136 features per a binding site in the DUD-E dataset. An example of what this looks like is shown in Additional File 1: Figure S1.

**Table 4:** CNN pharmacophore feature classification on ligand feature points

Pharmacophore feature	ROC-AUC
Aromatic	0.9821
HydrogenAcceptor	0.9586
HydrogenDonor	0.9514
Hydrophobic	0.9724
NegativeIon	0.9768
PositiveIon	0.9769

### RL models provide at least one good solution on the DUD-E diverse subset

The diverse subset of the DUD-E dataset is used to test the RL algorithm. This subset, provided by the developers of the DUD-E dataset, represents all the protein classes present in the dataset. For each system in the DUD-E dataset, all possible combinations of pharmacophore features from the cognate ligand are enumerated. Since the best possible *F*1 score differs from system to system, the *F*1 score normalized by the maximum possible *F*1 achievable from the ligand features is reported. We notice that the best *F*1 score across all systems are from pharmacophores that are either of size 3, 4, or 5; therefore, the mean of all possible pharmacophores of max size 5 are considered as a random selection baseline.

To evaluate the performance of a supervised learning approach for pharmacophore generation that ranks pharmacophore features individually, combinations of the top-3, top-4, and top-5 CNN ranked features are used as pharmacophores. Each of these pharmacophores, except one (*F*1 = 0.028), yield an *F*1 score of 0, indicating that a supervised approach trained on individual features is not sufficient for this problem and a RL approach is more ideal.

**Fig. 7:**
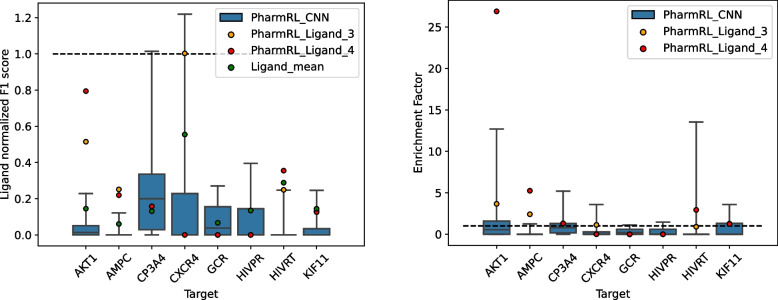
*F*1 scores divided by the max *F*1 score attainable from ligand features for RL models trained and tested on ligand derived features (PharmRL_Ligand) and all CNN features (PharmRL_CNN)

From this point onward, we will refer to the RL models trained on CNN features as PharmRL_CNN and the model trained on ligand features as PharmRL_Ligand. During training on the DUDE set, we observed that the models tend to converge on generating pharmacophores with only 3 features. This is likely due to the large number of actives in the DUDE dataset, which drives the model to prioritize enhancing recall performance. Because these pharmacophores lack selectivity, we also evaluate the models when they are required to generate pharmacophores with at least 4 features.

Figure 7 showcases the results of the pharmacophores generated from the application of our RL models on the 8 test systems. Each box plot is a culmination of 10 pharmacophores (minimum 3 and 4 features) from our CNN based models. The whiskers of the box plot are set to maximum and minimum value of the set. The performance of PharmRL_Ligand is also shown with a minimum of 3 (PharmRL_Ligand_3) and 4 features (PharmRL_Ligand_4). Ligand_mean is the *F*1 score obtained from the aforementioned random selection baseline. All the *F*1 scores are normalized by the max possible *F*1 score attainable from the ligand features. We also report other metrics for these models in Additional File 1: Table S1.

From Fig. 7, it is clear that for each system the models have generated a pharmacophore that does better than the average of random selection. The method consistently generate at least one pharmacophore that achieves an enrichment factor greater than 1, indicating that it performs better at identifying active compounds than random selection from the dataset. The model trained and tested on ligand features finds the best achievable solution in 2/8 systems indicating the model is capable of finding the right pharmacophore from ligand features on certain systems. For 5/8 systems, a model that uses the CNN predicted features are able to provide a pharmacophore that has better performance than the model trained on ligand-only features. Notably, for two of those systems (CP3A4 and CXCR4), the solutions provide a *F*1 score that is higher than that of the max *F*1 score achievable from the ligand-only features. This is empirical evidence that the CNN is able to predict pharmacophore features that are relevant in the context of the given binding site and could be used for molecular screening. The RL algorithm, however, is necessary to assemble pharmacophores in an automated way. We show an example of how PharmRl_Ligand selects features on the AKT1 cognate ligand with associated *Q*-scores in Additional File 1: Figure S2.

### RL models provide good pharmacophores for COVID Moonshot

To test the screening capability of the RL models on the SARS-CoV2 Mpro protein we used a dataset of 23 publicly released non-covalently bound protein-fragment structures [30]. The identified pharmacophore features from the 23 complexes are clustered together in 3D in the same manner as for the CNN features. We also generate CNN based pharmacophore features in the binding sites using one of the structures. We screen against the COVID Moonshot dataset and label active molecules as those molecules that have an IC$_{50}$ value < 5 μM. This evaluation was carried out in two retrospective phases. The “hit-to-lead” phase encompassed 979 molecules deposited before September 1st, 2020, of which 6% are considered actives [15]. The complete dataset, which represents the most up-to-date information, comprises 2062 molecules, of which 38% are considered actives.

**Fig. 8:**
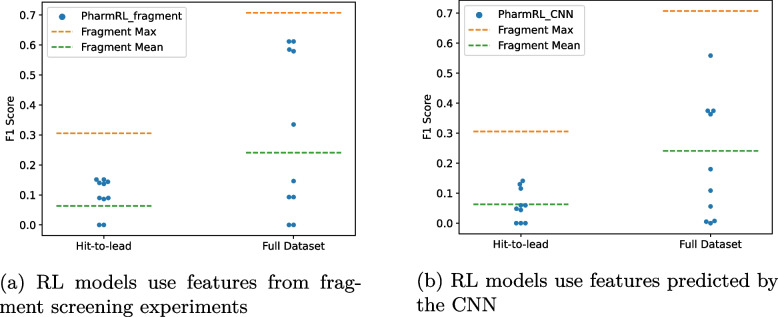
Performance of RL models on COVID screening experiments

Since it is computationally intractable to enumerate all possible pharmacophores from the fragment features, we sample 10,000 pharmacophores of sizes of 3, 4, or 5 and report the max and mean *F*1 score obtained (Fragment_Max and Fragment_Mean). We compare this to the performance of our RL models on fragment features (Fig. 8a) and CNN predicted features (Fig. 8b). It is important to emphasize here that the *F*1 scores presented in this experiment are the actual *F*1 scores and not max-normalized *F*1 scores. We also report other metrics for these models in Additional File 1: Tables S2, S3.

In both cases we can see that the RL models find pharmacophores that are close to the optimal *F*1 scores. This is exciting as it is an indication that the RL models can be used in tandem with pharmacophore features with “ground truth interactions” derived from experimentally determined fragment structure complexes. Furthermore, in some cases the RL models perform better than random for feature selection. Perhaps more exciting is that the CNN + RL framework was able to identify find good pharmacophores even in the absence of any fragment data. We provide example pharmacophores and further analysis in Additional File 1: Section S3.

### PharmRL has comparative performance to baselines on the LIT-PCBA dataset

Finally, the performance of PharmRL is evaluated and compared to Apo2ph4 [18] on the LIT-PCBA dataset. To perform this comparison, we directly use the pharmacophores provided by the Apo2ph4 authors. For a direct comparison, we also use the same PDB structures that were used by them for all the LIT-PCBA systems. Since their screening procedure involves proprietary software, we decided to create an open source benchmark using their pharmacophores. Therefore, we screen their pharmacophores on the LIT-PCBA dataset using pharmit with receptor exclusion turned on. The same parameters are used to screen our pharmacophores.

**Fig. 9:**
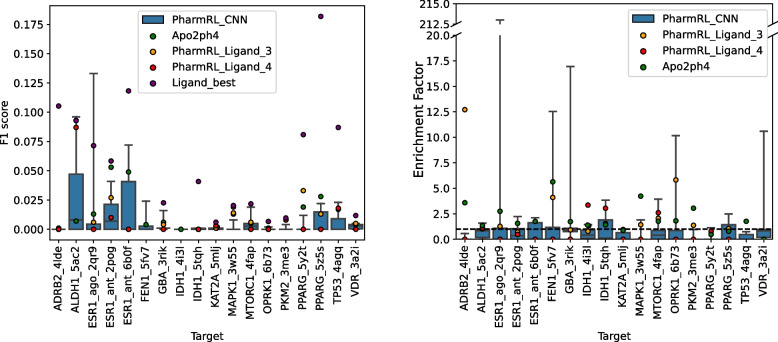
Performance of pharmacophore models on LIT-PCBA targets

Figure 9 provides the results of the pharmacophore models on the LIT-PCBA systems. The legend is consistent with the one defined beforehand in the “RL models provide at least one good solution on the DUD-E diverse subset” section. Note that we report actual *F*1 scores rather than ligand-normalized *F*1 scores. However, we do provide the best achievable *F*1 score based on ligand features, labeled as Ligand_best. Additional metrics for pharmacophore performance are reported in Additional File 1: Table S4.

From the figure, it is evident that PharmRL_CNN provides at least one pharmacophore that achieves a better *F*1 score and enrichment factor than Apo2ph4 on 12 out of 18 systems. On one of the systems (ESR1_ago), a pharmacophore even outperforms the best attainable performance based on ligand features, further indicating that the CNN provides relevant features for the RL models to screen. The method also yields pharmacophores with an enrichment factor greater than 1 for all systems except ADRB2, demonstrating that some of the pharmacophores possess significant screening strength.

## Discussion

In this work, we provide a framework to elucidate pharmacophores on a given binding pocket using only the protein structure. This is particularly important when co-crystal structures with cognate ligands do not exist. To accomplish this, we employ a CNN model to predict the potential locations of pharmacophore features within the binding site. Subsequently, these predictions are fed into an RL algorithm that utilizes a rotational equivariant neural network to generate pharmacophores that are subsets of these features.

The CNN model is trained using features extracted from co-crystal structures found in the PDBbind V. 2019 database. Since the prediction of pharmacophore features should rely solely on the local context surrounding a specific point, we input only the minimal local information into the CNN. The CNN demonstrates high accuracy in identifying the correct features at positions provided by the structures in the training set (Table 4). However, it needs to be retrained with adversarial samples to ensure that its predictions during the inference stage are physically plausible and relevant.

The CNN model can be considered a probe that identifies pharmacophore features in the binding site. However, ranking these points by themselves is not sufficient to form a valid pharmacophore for virtual screening. This is where the RL models come in as they are able to select a sufficient subset of them for succesful screening. In principle the RL models can also be used in conjunction with other methods that identify pharmacophore features on the binding site and our open source method supports that.

As demonstrated above (Figs. 7, 8, and 9), the pharmacophores generated using the CNN features and our RL model exhibit strong retrospective virtual screening performance, indicating the model’s ability to provide the correct features at relevant positions within the binding site. Additionally, in certain cases, as evidenced by the higher *F*1 score (systems CP3A4, and CXCR4 Fig. 7, Esr1_ago Fig. 9), the CNN can offer features that are more effective than those from the cognate ligand structures.

A challenge in pharmacophore methods development is the limited availability of virtual screening data to train models for distinguishing effective and ineffective pharmacophores. Additionally, the dataset may inherently contain biases regarding what constitutes a proficient pharmacophore within a binding pocket. For example, if a system predominantly consists of a congeneric series as its active molecules, relying solely on the *F*1 score can lead to skewed results, where highly rewarded pharmacophores may exclusively match molecules from that specific series. Furthermore, in the context of protein-ligand binding, a binding site has the potential to bind to multiple diverse types of ligands. Hence, there is no singular correct pharmacophore for a particular binding site. Consequently, this leads to substantial variability in the optimal policy for this task and thus a large variance in the policy learnt by the RL models. It is important to note that we do not expect great performance all the time even from a perfect model—the model may generate pharmacophores that match active molecules whose chemotypes are not present in a retrospective screening. Therefore, five RL models are trained to generate pharmacophores, with the aim of mitigating the inherent variability in the problem and accounting for potential biases in the dataset.

## Conclusions

As previously demonstrated, PharmRl consistently produce at least one effective pharmacophore for most test system in the dataset (refer to Figs. 7 and 8). It is important to emphasize that while PharmRL is compared against randomly selected ligand features, the ligand features are “ground truth” interaction points, making the pharmacophores generated from them inherently enriched. Finally, we also compare to an established baseline (Apo2ph4) on the LIT-PCBA dataset and show that PharmRL exhibits comparable performance to the baseline.

Our method offers notable advantages as it is completely open source and it has the capacity for human intervention. The sequential nature of the graph-building framework grants users the opportunity to decide which features should be included or excluded in the generated pharmacophore. Furthermore, users can control the size of the generated pharmacophore, enabling the addition or removal of nodes as required. Users could also incorporate features obtained from other methods such as fragment experiments and ensure that they are present in the generated pharmacophore. These user-friendly tools are implemented and easily accessible in a google colaboratory notebook (link) and the full open source code for training and inference is available at https://github.com/RishalAggarwal/Pharmrl.

## Supplementary information

Additional file 1. PharmRL: Pharmacophore elucidation with Deep Geometric Reinforcement Learning, Figures S1-S3, Tables S1-S4. Figure S1. CNN pharmacophore feature points predicted for a binding site. Figure S2. Pharmacophore selection process shown on the Serine/threonine-protein kinase cognate ligandusing the PharmRL trained on ligand features. Figure S3. Example pharmacophores generated using features top ranked by the CNN and those selected by the RL model on the COVID moonshot dataset. Table S1. Results of Model Runs on DUD-E test set. Table S2. PharmRL performance on Covid Moonshot on using features obtained from crystal structures of bound fragments. Table S3. PharmRL performance on Covid Moonshot on using features obtained from the CNN. Table S4. Performance of RL models and Apo2ph4 on LIT-PCBA systems.

## Data Availability

No datasets were generated or analyzed during the current study.

## Abbreviations

CNN: Convolutional neural networks
DUD-E: Dataset of Useful Decoys - Enhanced
RL: Reinforcement learning
MDP: Markov Decision process
