# Supplementary material for: PharmRL: pharmacophore elucidation with deep geometric reinforcement learning
Source: BMC Biol. 2024 Dec 31;22:301. doi: 10.1186/s12915-024-02096-5 (PMC11687028; doi:10.1186/s12915-024-02096-5)
Supplement: Supplementary file 1 — Additional file 1. PharmRL: Pharmacophore elucidation with Deep Geometric Reinforcement Learning, Figures S1-S3, Tables S1-S4. Figure S1. CNN pharmacophore feature points predicted for a binding site. Figure S2. Pharmacophore selection process shown on the Serine/threonine-protein kinase cognate ligandusing the PharmRL trained on ligand features. Figure S3. Example pharmacophores generated using features top ranked by the CNN and those selected by the RL model on the COVID moonshot dataset. Table S1. Results of Model Runs on DUD-E test set. Table S2. PharmRL performance on Covid Moonshot on using features obtained from crystal structures of bound fragments. Table S3. PharmRL performance on Covid Moonshot on using features obtained from the CNN. Table S4. Performance of RL models and Apo2ph4 on LIT-PCBA systems. [file 12915_2024_2096_MOESM1_ESM.pdf]

Supporting Information

PharmRL: Pharmacophore elucidation with Deep Geometric Reinforcement Learning

Rishal Aggarwal[1,2] and David R. Koes[2*]

[1]Joint PhD Program in Computational Biology, Carnegie Mellon University-University of Pittsburgh, Pittsburgh, Pennsylvania.
[2]Computational & Systems Biology, University of Pittsburgh, Pittsburgh, Pennsylvania.

*Corresponding author(s). E-mail(s): dkoes@pitt.edu;
Contributing authors: rishal.aggarwal@pitt.edu;

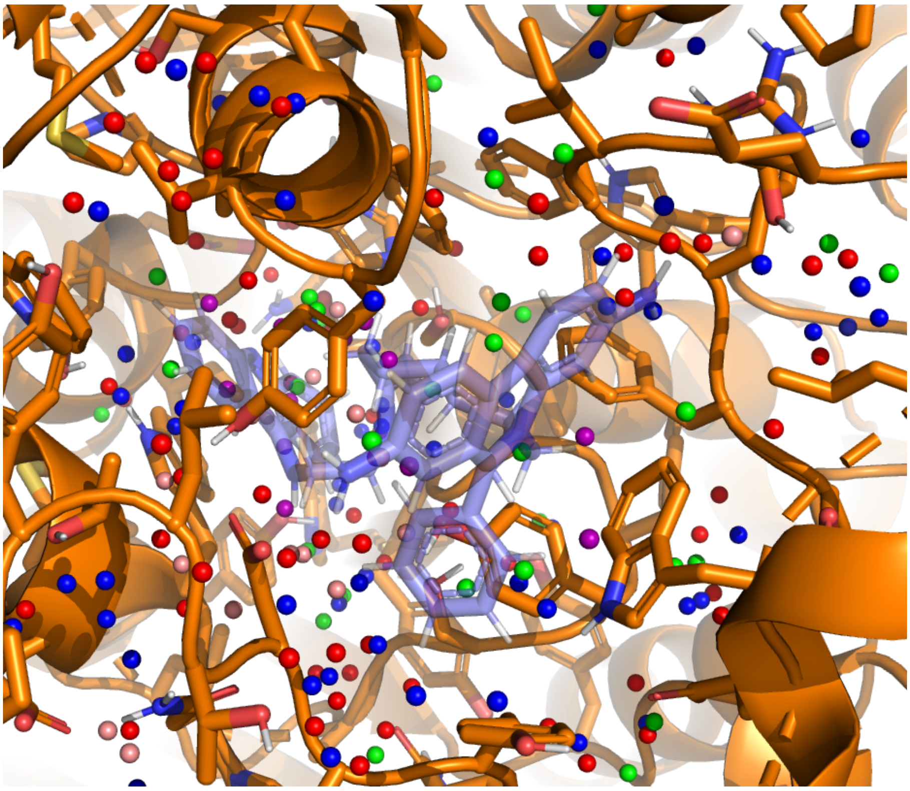

Fig. S1: All pharmacophore feature points predicted for a binding site. The colors are as follows: Aromatic - Purple, HydrogenAcceptor - Blue, HydrogenDonor - Red, Hydrophobic - Green, NegativeIon - SkyBlue, PositiveIon - DeepSalmon

S1 Example of CNN features on the binding site

Figure S1 shows an example system with all the predicted pharmacophore feature points for all the six classes.

S2 Pharmacophore selection by RL algorithm

Figure S2 shows an example of how an RL model selects features from a set to form the pharmacophore. In this example, it selects 4 features by greedily picking the feature that maximizes the Q score at each step of the pharmacophore formation. The model was run in the "min_4" setting and it has settled with selecting just 4 features.

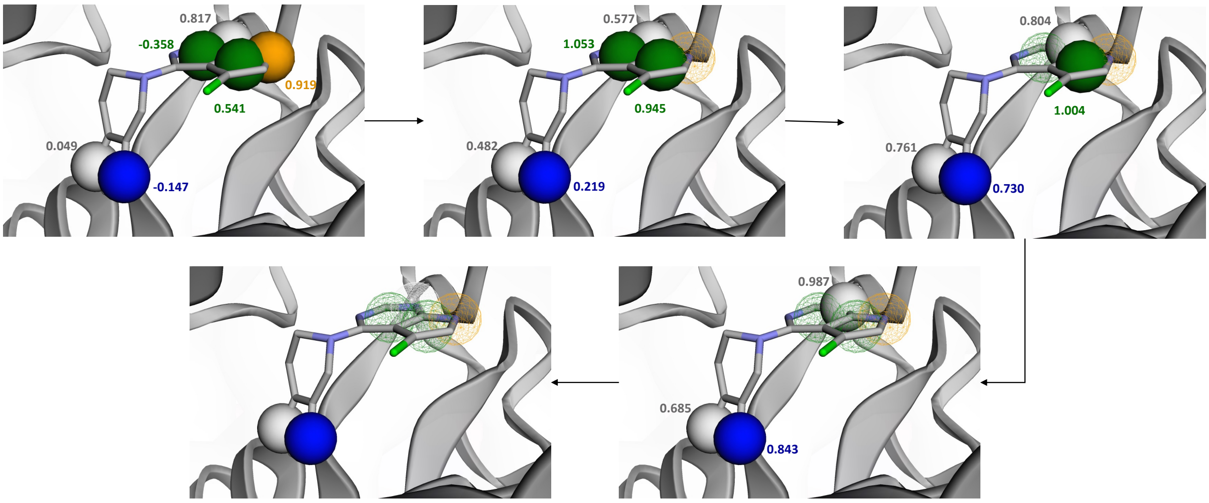

Fig. S2: Pharmacophore selection process shown on the Serine/threonine-protein kinase cognate ligand (PDB ID AKT1) using the model trained on ligand features. Meshed spheres represent selected features and solid spheres represent query features. The numbers represent Q-scores associated with the features.

S3 Example Pharmacophores

In Figure S3, we present example pharmacophores generated using our RL method, with fragment-based features on the left and CNN-derived features on the right. These are compared with the pharmacophore

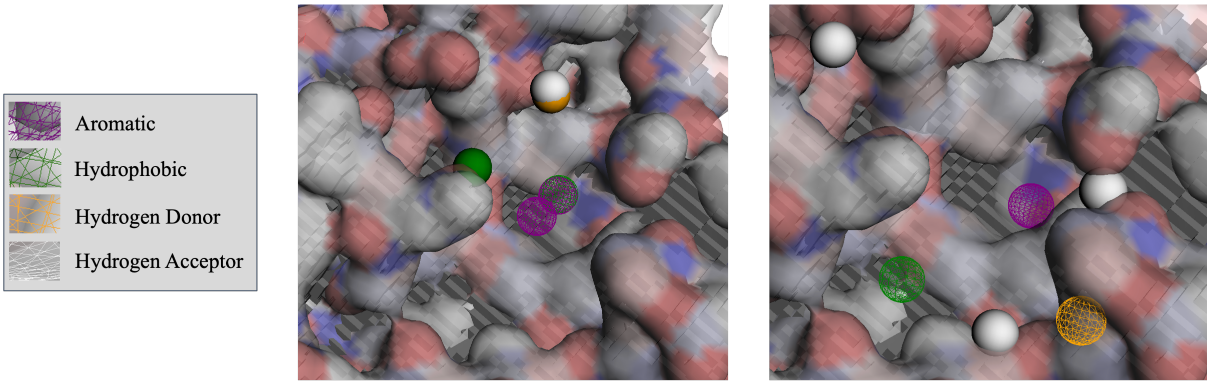

Fig. S3: Example pharmacophores generated using features top ranked by the CNN (bold spheres) and those selected by the RL model (mesh spheres) on the COVID moonshot dataset. The pharmacophores are based on features extracted from bound fragments (left) and CNN (right).

created by utilizing the top 3 features ranked by the CNN, which are shown as solid spheres versus the mesh spheres of the pharmacophore generated from the RL model..

Interestingly, both pharmacophores formed using CNN-ranked features result in an F1 score of 0. This is more evident in the case of the CNN-derived features (right), where the top-ranked features are widely dispersed across the binding site, making it possible for only large molecules to match with them. In contrast, the top-ranked features from the fragment experiments are much closer to each other, yet the pharmacophore still returns an F1 score of 0. Consequently, we can conclude that an approach that individually ranks features is unsuitable for the automated generation of a pharmacophore.

An intriguing observation regarding both pharmacophores generated by the RL algorithm is that they both support the existence of an aromatic group within the same region of the binding site. This demonstrates a degree of agreement between the two pharmacophores. The pharmacophore generated by the RL model that uses CNN features, however, offers a more diverse set of features that could be useful for screening molecules.

S4 RL performance scores

In Table S1 we list out results of all the pharmacophore models applied on the 8 test systems from the DUD-E dataset. Table S2 reports performance achieved by the models on the COVID moonshot dataset when using pharmacophore features obtained from fragment screening data. Table S3 reports model performance when we use features obtained from the CNN instead. Finally the results for the pharmacophore models and Apo2ph4 on LIT-PCBA systems are reported in Table S4. In all the tables, model_ligand represents the model trained and inferred on ligand features, while model_cnn_# represents the model trained and tested on CNN features.

Table S1: Results of Model Runs on DUD-E test set

System	Model	Min_Feats	Precision	Recall	F1	Enrichment	Hit_rate	Num_Feats	Guner-Henry
AKT1	best_ligand	3	0.291	0.181	0.223	16.641	0.011	4	0.262
	model_ligand	3	0.064	0.546	0.115	3.669	0.149	3	0.158
		4	0.471	0.109	0.177	26.891	0.004	4	0.379
	model_cnn_1	3	0.000	0.000	0.000	0.000	0.006	3	0.000
		4	0.000	0.000	0.000	0.000	0.004	4	0.000
	model_cnn_2	3	0.068	0.041	0.051	3.874	0.011	3	0.060
		4	0.000	0.000	0.000	0.000	0.000	4	0.000
	model_cnn_3	3	0.019	0.102	0.033	1.111	0.092	3	0.036
		4	0.000	0.000	0.000	0.000	0.000	4	0.000
	model_cnn_4	3	0.028	0.003	0.006	1.587	0.002	4	0.022
		4	0.028	0.003	0.006	1.587	0.002	4	0.022
	model_cnn_5	3	0.222	0.007	0.013	12.699	0.001	3	0.168
		4	0.000	0.000	0.000	0.000	0.000	4	0.000
AMPC	best_ligand	3	0.333	0.208	0.256	20.125	0.010	3	0.300
	model_ligand	3	0.040	0.167	0.065	2.415	0.069	3	0.067
		4	0.087	0.042	0.056	5.250	0.008	4	0.075
	model_cnn_1	3	0.000	0.000	0.000	0.000	0.001	3	0.000
		4	0.000	0.000	0.000	0.000	0.000	4	0.000
	model_cnn_2	3	0.000	0.000	0.000	0.000	0.001	3	0.000
		4	0.000	0.000	0.000	0.000	0.000	4	0.000
	model_cnn_3	3	0.000	0.000	0.000	0.000	0.000	4	0.000
		4	0.000	0.000	0.000	0.000	0.000	4	0.000
	model_cnn_4	3	0.000	0.000	0.000	0.000	0.002	3	0.000
		4	0.000	0.000	0.000	0.000	0.000	4	0.000
	model_cnn_5	3	0.021	0.062	0.031	1.258	0.050	3	0.030
		4	0.000	0.000	0.000	0.000	0.014	4	0.000
CP3A4	best_ligand	3	0.094	0.088	0.091	6.643	0.013	4	0.092
	model_ligand	3	0.019	0.012	0.014	1.304	0.009	4	0.017
		4	0.019	0.012	0.014	1.304	0.009	4	0.017
	model_cnn_1	3	0.030	0.271	0.054	2.111	0.128	3	0.079
		4	0.074	0.124	0.093	5.207	0.024	4	0.084
	model_cnn_2	3	0.000	0.000	0.000	0.000	0.000	4	0.000
		4	0.000	0.000	0.000	0.000	0.000	4	0.000
	model_cnn_3	3	0.009	0.012	0.010	0.658	0.018	4	0.010
		4	0.009	0.012	0.010	0.658	0.018	4	0.010
	model_cnn_4	3	0.016	0.082	0.027	1.115	0.074	3	0.030
		4	0.000	0.000	0.000	0.000	0.002	4	0.000
	model_cnn_5	3	0.013	0.824	0.026	0.930	0.886	3	0.025
		4	0.019	0.094	0.032	1.354	0.070	4	0.035
CXCR4	best_ligand	3	0.013	0.675	0.026	1.125	0.600	3	0.072
	model_ligand	3	0.013	0.675	0.026	1.128	0.598	3	0.072
		4	0.000	0.000	0.000	0.000	0.024	4	0.000
	model_cnn_1	3	0.000	0.000	0.000	0.000	0.000	4	0.000
		4	0.000	0.000	0.000	0.000	0.000	4	0.000
	model_cnn_2	3	0.000	0.000	0.000	0.000	0.002	3	0.000
		4	0.000	0.000	0.000	0.000	0.000	4	0.000
	model_cnn_3	3	0.012	0.375	0.024	1.056	0.355	3	0.066
		4	0.000	0.000	0.000	0.000	0.006	4	0.000
	model_cnn_4	3	0.005	0.025	0.008	0.399	0.063	3	0.009
		4	0.000	0.000	0.000	0.000	0.015	4	0.000
	model_cnn_5	3	0.042	0.025	0.031	3.590	0.007	3	0.037
		4	0.000	0.000	0.000	0.000	0.001	4	0.000
GCR	best_ligand	3	0.113	0.147	0.128	6.688	0.022	4	0.119
	model_ligand	3	0.000	0.000	0.000	0.000	0.000	4	0.000
		4	0.000	0.000	0.000	0.000	0.000	4	0.000
	model_cnn_1	3	0.010	0.167	0.019	0.604	0.276	3	0.036
		4	0.000	0.000	0.000	0.000	0.001	4	0.000
	model_cnn_2	3	0.000	0.000	0.000	0.000	0.021	3	0.000
		4	0.000	0.000	0.000	0.000	0.000	4	0.000
	model_cnn_3	3	0.015	0.674	0.030	0.910	0.741	3	0.046
		4	0.010	0.422	0.020	0.610	0.693	4	0.034
	model_cnn_4	3	0.000	0.000	0.000	0.000	0.000	4	0.000
		4	0.000	0.000	0.000	0.000	0.000	4	0.000
	model_cnn_5	3	0.019	0.233	0.035	1.104	0.211	3	0.057
		4	0.006	0.019	0.010	0.375	0.052	4	0.009

System	Model	Min_Feats	Precision	Recall	F1	Enrichment	Hit_rate	Num_Feats	Guner-Henry
HIVPR	best_ligand	3	0.079	0.168	0.107	5.345	0.031	4	0.098
	model_ligand	3	0.000	0.000	0.000	0.000	0.008	5	0.000
		4	0.000	0.000	0.000	0.000	0.008	5	0.000
	model_cnn_1	3	0.000	0.000	0.000	0.000	0.000	4	0.000
		4	0.000	0.000	0.000	0.000	0.000	4	0.000
	model_cnn_2	3	0.000	0.000	0.000	0.000	0.001	3	0.000
		4	0.000	0.000	0.000	0.000	0.000	4	0.000
	model_cnn_3	3	0.018	0.828	0.035	1.218	0.680	3	0.071
		4	0.000	0.000	0.000	0.000	0.001	4	0.000
	model_cnn_4	3	0.022	0.728	0.042	1.479	0.492	3	0.101
		4	0.000	0.000	0.000	0.000	0.000	4	0.000
	model_cnn_5	3	0.012	0.099	0.021	0.786	0.126	3	0.029
		4	0.000	0.000	0.000	0.000	0.021	4	0.000
HIVRT	best_ligand	3	0.163	0.086	0.112	9.269	0.009	4	0.143
	model_ligand	3	0.016	0.112	0.028	0.909	0.124	3	0.035
		4	0.052	0.033	0.040	2.938	0.011	4	0.046
	model_cnn_1	3	0.015	0.080	0.025	0.849	0.094	3	0.028
		4	0.000	0.000	0.000	0.000	0.000	4	0.000
	model_cnn_2	3	0.238	0.015	0.028	13.545	0.001	3	0.182
		4	0.000	0.000	0.000	0.000	0.000	4	0.000
	model_cnn_3	3	0.000	0.000	0.000	0.000	0.002	3	0.000
		4	0.000	0.000	0.000	0.000	0.000	6	0.000
	model_cnn_4	3	0.000	0.000	0.000	0.000	0.006	3	0.000
		4	0.000	0.000	0.000	0.000	0.000	4	0.000
	model_cnn_5	3	0.000	0.000	0.000	0.000	0.001	3	0.000
		4	0.000	0.000	0.000	0.000	0.000	4	0.000
KIF11	best_ligand	3	0.500	0.233	0.318	30.026	0.008	5	0.431
	model_ligand	3	0.021	0.793	0.041	1.277	0.621	3	0.082
		4	0.021	0.793	0.040	1.233	0.643	4	0.077
	model_cnn_1	3	0.000	0.000	0.000	0.000	0.001	3	0.000
		4	0.000	0.000	0.000	0.000	0.001	4	0.000
	model_cnn_2	3	0.000	0.000	0.000	0.000	0.002	3	0.000
		4	0.000	0.000	0.000	0.000	0.001	4	0.000
	model_cnn_3	3	0.060	0.112	0.078	3.598	0.031	3	0.071
		4	0.000	0.000	0.000	0.000	0.000	4	0.000
	model_cnn_4	3	0.000	0.000	0.000	0.000	0.000	3	0.000
		4	0.000	0.000	0.000	0.000	0.000	4	0.000
	model_cnn_5	3	0.029	0.474	0.055	1.762	0.269	3	0.103
		4	0.045	0.009	0.014	2.730	0.003	4	0.036

Table S2: PharmRL performance on Covid Moonshot on using features obtained from crystal structures of bound fragments.

System	Model	Min_Feats	Precision	Recall	F1	Enrichment	Hit_rate	Num_Feats	Guner-Henry
Hit-to-lead	Best sampled	3	0.208	0.450	0.284	4.171	0.108	4	0.244
	model_cnn_1	3	0.084	0.783	0.151	1.370	0.572	4	0.114
		4	0.084	0.783	0.151	1.370	0.572	4	0.114
	model_cnn_2	3	0.076	0.867	0.140	1.247	0.695	3	0.087
		4	0.112	0.200	0.144	1.834	0.109	4	0.120
	model_cnn_3	3	0.068	0.133	0.090	1.108	0.120	4	0.074
		4	0.068	0.133	0.090	1.108	0.120	4	0.074
	model_cnn_4	3	0.077	0.583	0.137	1.266	0.461	3	0.112
		4	0.056	0.183	0.086	0.922	0.199	4	0.071
	model_cnn_5	3	0.000	0.000	0.000	0.000	0.000	3	0.000
		4	0.000	0.000	0.000	0.000	0.000	4	0.000
Full dataset	Best sampled	3	0.605	0.797	0.688	1.490	0.535	4	0.421
	model_cnn_1	3	0.451	0.950	0.612	1.110	0.856	4	0.120
		4	0.451	0.950	0.612	1.110	0.856	4	0.120
	model_cnn_2	3	0.425	0.908	0.579	1.046	0.868	3	0.087
		4	0.296	0.097	0.146	0.728	0.133	4	0.207
	model_cnn_3	3	0.299	0.055	0.093	0.735	0.075	4	0.217
		4	0.299	0.055	0.093	0.735	0.075	4	0.217
	model_cnn_4	3	0.454	0.820	0.585	1.119	0.733	3	0.178
		4	0.371	0.305	0.335	0.912	0.335	4	0.229
	model_cnn_5	3	0.000	0.000	0.000	0.000	0.000	3	0.000
		4	0.000	0.000	0.000	0.000	0.000	4	0.000

Table S3: PharmRL performance on Covid Moonshot on using features obtained from the CNN

System	Model	Min_Feats	Precision	Recall	F1	Enrichment	Hit_rate	Num_Feats	Guner-Henry
Hit-to-lead	Best sampled	3	0.208	0.450	0.284	4.171	0.108	4	0.244
	model_cnn_1	3	0.081	0.200	0.115	1.326	0.151	3	0.094
		4	0.000	0.000	0.000	0.000	0.007	4	0.000
	model_cnn_2	3	0.065	0.033	0.044	1.055	0.032	3	0.055
		4	0.000	0.000	0.000	0.000	0.005	4	0.000
	model_cnn_3	3	0.087	0.033	0.048	1.422	0.023	3	0.072
		4	0.000	0.000	0.000	0.000	0.000	4	0.000
	model_cnn_4	3	0.073	0.050	0.059	1.196	0.042	4	0.065
		4	0.073	0.050	0.059	1.196	0.042	4	0.065
	model_cnn_5	3	0.077	0.800	0.141	1.264	0.633	3	0.097
		4	0.146	0.117	0.130	2.384	0.049	4	0.132
Full dataset	Best sampled	3	0.605	0.797	0.688	1.490	0.535	4	0.421
	model_cnn_1	3	0.551	0.271	0.363	1.356	0.200	3	0.408
		4	0.118	0.003	0.005	0.290	0.009	4	0.088
	model_cnn_2	3	0.372	0.119	0.180	0.916	0.130	3	0.266
		4	0.086	0.004	0.007	0.211	0.018	4	0.063
	model_cnn_3	3	0.239	0.070	0.109	0.589	0.119	3	0.167
		4	0.000	0.000	0.000	0.000	0.000	4	0.000
	model_cnn_4	3	0.589	0.275	0.375	1.450	0.189	4	0.444
		4	0.589	0.275	0.375	1.450	0.189	4	0.444
	model_cnn_5	3	0.422	0.828	0.559	1.038	0.797	3	0.117
		4	0.312	0.031	0.056	0.767	0.040	4	0.230

Table S4: Performance of RL models and Apo2ph4 on LIT-PCBA systems

Target	system	model	Min_Feats	precision	recall	f1	enrichment	hit_rate	Num_Feats	Guner-Henry
ADRB2	4LDE	Apo2ph4	3	0.000	0.412	0.000	3.585	0.115	3	0.091
		best_ligand	3	0.500	0.059	0.105	9185.176	0.000	5	0.390
		model_cnn_1	3	0.000	0.000	0.000	0.000	0.030	3	0.000
			4	0.000	0.000	0.000	0.000	0.000	4	0.000
		model_cnn_2	3	0.000	0.000	0.000	0.000	0.000	4	0.000
			4	0.000	0.000	0.000	0.000	0.000	4	0.000
		model_cnn_3	3	0.000	0.000	0.000	0.000	0.000	4	0.000
			4	0.000	0.000	0.000	0.000	0.000	4	0.000
		model_cnn_4	3	0.000	0.118	0.000	0.571	0.206	3	0.023
			4	0.000	0.000	0.000	0.000	0.000	4	0.000
		model_cnn_5	3	0.000	0.000	0.000	0.000	0.284	3	0.000
			4	0.000	0.000	0.000	0.000	0.084	4	0.000
		model_ligand	3	0.001	0.176	0.001	12.710	0.014	3	0.044
			4	0.000	0.000	0.000	0.000	0.000	4	0.000
ALDH1	5AC2	Apo2ph4	3	0.067	0.003	0.007	1.347	0.003	6	0.051
		best_ligand	3	0.050	0.607	0.093	1.016	0.598	3	0.076
		model_cnn_1	3	0.034	0.000	0.000	0.697	0.000	3	0.026
			4	0.000	0.000	0.000	0.000	0.000	4	0.000
		model_cnn_2	3	0.044	0.010	0.017	0.899	0.012	3	0.036
			4	0.000	0.000	0.000	0.000	0.000	4	0.000
		model_cnn_3	3	0.047	0.108	0.066	0.955	0.113	3	0.055
			4	0.000	0.000	0.000	0.000	0.000	6	0.000
		model_cnn_4	3	0.053	0.544	0.096	1.068	0.509	3	0.087
			4	0.079	0.003	0.006	1.587	0.002	4	0.060
		model_cnn_5	3	0.039	0.105	0.057	0.794	0.132	3	0.048
			4	0.050	0.006	0.010	1.016	0.006	4	0.039
		model_ligand	3	0.051	0.573	0.093	1.021	0.561	3	0.080
			4	0.054	0.215	0.087	1.100	0.195	4	0.076
ESR1_ago	2QR9	Apo2ph4	3	0.006	0.231	0.013	2.748	0.084	4	0.057
		best_ligand	3	0.047	0.154	0.071	19.814	0.008	4	0.073
		model_cnn_1	3	0.000	0.000	0.000	0.000	0.000	4	0.000
			4	0.000	0.000	0.000	0.000	0.000	4	0.000
		model_cnn_2	3	0.005	0.154	0.009	2.078	0.074	3	0.039
			4	0.500	0.077	0.133	213.000	0.000	4	0.394
		model_cnn_3	3	0.002	0.154	0.003	0.705	0.218	3	0.031
			4	0.000	0.000	0.000	0.000	0.001	4	0.000
		model_cnn_4	3	0.000	0.000	0.000	0.000	0.008	3	0.000
			4	0.000	0.000	0.000	0.000	0.000	6	0.000
		model_cnn_5	3	0.003	0.077	0.005	1.190	0.065	3	0.020
			4	0.000	0.000	0.000	0.000	0.007	4	0.000
		model_ligand	3	0.003	0.615	0.006	1.272	0.484	3	0.081
			4	0.000	0.000	0.000	0.000	0.014	4	0.000
ESR1_ant	2POG	Apo2ph4	3	0.032	0.157	0.053	1.565	0.100	4	0.057
		best_ligand	3	0.030	0.745	0.058	1.488	0.501	3	0.105
		model_cnn_1	3	0.000	0.000	0.000	0.000	0.000	5	0.000
			4	0.000	0.000	0.000	0.000	0.000	5	0.000
		model_cnn_2	3	0.022	0.010	0.014	1.066	0.009	3	0.019
			4	0.000	0.000	0.000	0.000	0.000	4	0.000
		model_cnn_3	3	0.019	0.176	0.034	0.937	0.188	3	0.047
			4	0.000	0.000	0.000	0.000	0.001	4	0.000
		model_cnn_4	3	0.015	0.059	0.023	0.717	0.082	3	0.024
			4	0.000	0.000	0.000	0.000	0.001	4	0.000
		model_cnn_5	3	0.035	0.049	0.041	1.738	0.028	3	0.038
			4	0.045	0.010	0.016	2.228	0.004	4	0.036
		model_ligand	3	0.015	0.147	0.027	0.714	0.206	3	0.038
			4	0.010	0.010	0.010	0.476	0.021	4	0.010

Target	system	model	Min_Feats	precision	recall	f1	enrichment	hit_rate	Num_Feats	Guner-Henry
ESR1_ant	6B0F	Apo2ph4	3	0.036	0.078	0.049	1.751	0.045	4	0.044
		best_ligand	3	0.067	0.490	0.118	3.294	0.149	3	0.148
		model_cnn_1	3	0.000	0.000	0.000	0.000	0.000	4	0.000
			4	0.000	0.000	0.000	0.000	0.000	4	0.000
		model_cnn_2	3	0.042	0.245	0.072	2.067	0.119	3	0.082
			4	0.043	0.176	0.069	2.116	0.083	4	0.070
		model_cnn_3	3	0.021	0.157	0.037	1.028	0.153	3	0.047
			4	0.000	0.000	0.000	0.000	0.000	5	0.000
		model_cnn_4	3	0.000	0.000	0.000	0.000	0.000	3	0.000
			4	0.000	0.000	0.000	0.000	0.000	4	0.000
		model_cnn_5	3	0.037	0.049	0.042	1.816	0.027	3	0.039
			4	0.000	0.000	0.000	0.000	0.001	4	0.000
		model_ligand	3	0.000	0.000	0.000	0.000	0.000	4	0.000
			4	0.000	0.000	0.000	0.000	0.000	4	0.000
FEN1	5FV7	Apo2ph4	3	0.002	0.057	0.004	1.764	0.032	5	0.015
		model_cnn_1	3	0.000	0.000	0.000	0.000	0.000	3	0.000
			4	0.000	0.000	0.000	0.000	0.000	4	0.000
		model_cnn_2	3	0.000	0.000	0.000	0.000	0.000	4	0.000
			4	0.000	0.000	0.000	0.000	0.000	4	0.000
		model_cnn_3	3	0.013	0.165	0.024	12.536	0.013	3	0.050
			4	0.000	0.000	0.000	0.000	0.000	4	0.000
		model_cnn_4	3	0.001	0.263	0.002	1.003	0.262	3	0.049
			4	0.007	0.019	0.010	6.411	0.003	4	0.010
		model_cnn_5	3	0.001	0.314	0.003	1.235	0.255	3	0.059
			4	0.000	0.000	0.000	0.000	0.000	4	0.000
GBA	3RIK	Apo2ph4	3	0.003	0.054	0.006	5.633	0.010	5	0.016
		best_ligand	3	0.030	0.018	0.023	54.001	0.000	4	0.027
		model_cnn_1	3	0.001	0.108	0.001	0.927	0.117	3	0.024
			4	0.000	0.000	0.000	0.000	0.002	4	0.000
		model_cnn_2	3	0.000	0.373	0.001	0.733	0.510	3	0.046
			4	0.000	0.000	0.000	0.000	0.000	4	0.000
		model_cnn_3	3	0.001	0.139	0.001	1.122	0.123	3	0.031
			4	0.010	0.048	0.016	16.931	0.003	4	0.019
		model_cnn_4	3	0.001	0.458	0.001	0.895	0.511	3	0.056
			4	0.000	0.295	0.001	0.880	0.335	4	0.049
		model_cnn_5	3	0.001	0.416	0.001	0.948	0.439	3	0.059
			4	0.001	0.175	0.003	2.635	0.066	4	0.042
		model_ligand	3	0.002	0.036	0.004	4.103	0.009	3	0.011
			4	0.000	0.000	0.000	0.000	0.000	4	0.000
IDH1	4I3L	Apo2ph4	3	0.000	0.000	0.000	0.000	0.000	5	0.000
		model_cnn_1	3	0.000	0.051	0.000	0.268	0.191	3	0.010
			4	0.000	0.026	0.000	1.446	0.018	4	0.006
		model_cnn_2	3	0.000	0.026	0.000	0.354	0.072	3	0.006
			4	0.000	0.000	0.000	0.000	0.000	4	0.000
		model_cnn_3	3	0.000	0.154	0.000	0.903	0.170	3	0.032
			4	0.000	0.000	0.000	0.000	0.001	4	0.000
		model_cnn_4	3	0.000	0.436	0.000	0.886	0.492	3	0.055
			4	0.000	0.051	0.000	0.478	0.107	4	0.011
		model_cnn_5	3	0.000	0.026	0.000	1.121	0.023	3	0.006
			4	0.000	0.000	0.000	0.000	0.000	4	0.000

Target	system	model	Min_Feats	precision	recall	f1	enrichment	hit_rate	Num_Feats	Guner-Henry
IDH1	5TQH	Apo2ph4	3	0.000	0.231	0.000	1.731	0.133	4	0.050
		best_ligand	3	0.100	0.026	0.041	927.779	0.000	5	0.081
		model_cnn_1	3	0.000	0.000	0.000	0.000	0.000	3	0.000
			4	0.000	0.000	0.000	0.000	0.000	4	0.000
		model_cnn_2	3	0.000	0.026	0.000	0.223	0.115	3	0.006
			4	0.000	0.026	0.001	3.840	0.007	4	0.007
		model_cnn_3	3	0.000	0.128	0.001	3.065	0.042	3	0.031
			4	0.000	0.000	0.000	0.000	0.000	5	0.000
		model_cnn_4	3	0.000	0.000	0.000	0.000	0.000	5	0.000
			4	0.000	0.000	0.000	0.000	0.000	5	0.000
		model_cnn_5	3	0.000	0.256	0.000	0.556	0.461	3	0.035
			4	0.000	0.128	0.001	2.338	0.055	4	0.030
		model_ligand	3	0.000	0.564	0.000	0.919	0.614	3	0.054
			4	0.000	0.000	0.000	0.000	0.017	4	0.000
KAT2A	5MLJ	Apo2ph4	3	0.001	0.057	0.002	1.387	0.041	5	0.014
			4	0.001	0.139	0.001	1.324	0.105	4	0.032
		best_ligand	3	0.004	0.010	0.006	7.691	0.001	4	0.006
		model_cnn_1	3	0.000	0.000	0.000	0.000	0.000	4	0.000
			4	0.000	0.000	0.000	0.000	0.000	4	0.000
		model_cnn_2	3	0.001	0.021	0.001	0.983	0.021	3	0.005
			4	0.000	0.005	0.001	0.658	0.008	4	0.002
		model_cnn_3	3	0.000	0.304	0.001	0.629	0.484	3	0.039
			4	0.000	0.000	0.000	0.000	0.002	4	0.000
		model_cnn_4	3	0.000	0.031	0.001	0.674	0.046	3	0.008
			4	0.000	0.000	0.000	0.000	0.000	4	0.000
		model_cnn_5	3	0.000	0.000	0.000	0.000	0.000	3	0.000
			4	0.000	0.000	0.000	0.000	0.000	4	0.000
		model_ligand	3	0.000	0.216	0.001	0.783	0.276	3	0.039
			4	0.002	0.005	0.003	3.351	0.002	5	0.003
MAPK1	3W55	Apo2ph4	3	0.007	0.182	0.013	1.420	0.128	5	0.044
		best_ligand	3	0.047	0.013	0.020	9.466	0.001	5	0.038
		model_cnn_1	3	0.000	0.000	0.000	0.000	0.000	3	0.000
			4	0.000	0.000	0.000	0.000	0.000	5	0.000
		model_cnn_2	3	0.000	0.000	0.000	0.000	0.001	3	0.000
			4	0.000	0.000	0.000	0.000	0.000	4	0.000
		model_cnn_3	3	0.000	0.000	0.000	0.000	0.000	3	0.000
			4	0.000	0.000	0.000	0.000	0.000	4	0.000
		model_cnn_4	3	0.000	0.000	0.000	0.000	0.000	3	0.000
			4	0.000	0.000	0.000	0.000	0.000	4	0.000
		model_cnn_5	3	0.005	0.016	0.008	1.109	0.015	3	0.008
			4	0.009	0.003	0.005	1.902	0.002	4	0.008
		model_ligand	3	0.008	0.078	0.014	1.546	0.050	3	0.024
			4	0.015	0.026	0.019	3.043	0.009	4	0.018
MTORC1	4FAP	Apo2ph4	3	0.003	0.773	0.005	0.878	0.881	4	0.023
		best_ligand	3	0.017	0.031	0.022	5.713	0.005	3	0.020
		model_cnn_1	3	0.000	0.000	0.000	0.000	0.000	3	0.000
			4	0.000	0.000	0.000	0.000	0.000	4	0.000
		model_cnn_2	3	0.012	0.052	0.019	3.936	0.013	3	0.021
			4	0.000	0.000	0.000	0.000	0.000	5	0.000
		model_cnn_3	3	0.003	0.144	0.005	0.857	0.168	3	0.032
			4	0.007	0.041	0.013	2.525	0.016	4	0.016
		model_cnn_4	3	0.002	0.206	0.005	0.824	0.250	3	0.040
			4	0.000	0.000	0.000	0.000	0.001	4	0.000
		model_cnn_5	3	0.003	0.588	0.005	0.864	0.680	3	0.048
			4	0.000	0.000	0.000	0.000	0.000	4	0.000
		model_ligand	3	0.003	0.216	0.006	0.960	0.226	3	0.044
			4	0.000	0.000	0.000	0.000	0.000	5	0.000

Target	system	model	Min_Feats	precision	recall	f1	enrichment	hit_rate	Num_Feats	Guner-Henry
OPRK1	6B73	Apo2ph4	3	0.000	0.083	0.001	4.229	0.020	5	0.021
		best_ligand	3	0.004	0.042	0.007	40.863	0.001	5	0.013
		model_cnn_1	3	0.000	0.000	0.000	0.000	0.031	3	0.000
			4	0.000	0.000	0.000	0.000	0.000	4	0.000
		model_cnn_2	3	0.000	0.000	0.000	0.000	0.006	3	0.000
			4	0.000	0.000	0.000	0.000	0.001	4	0.000
		model_cnn_3	3	0.000	0.125	0.000	0.461	0.271	3	0.023
			4	0.000	0.042	0.000	0.989	0.042	4	0.010
		model_cnn_4	3	0.000	0.292	0.000	1.496	0.195	3	0.059
			4	0.000	0.000	0.000	0.000	0.009	4	0.000
		model_cnn_5	3	0.001	0.042	0.002	10.170	0.004	3	0.011
			4	0.000	0.000	0.000	0.000	0.000	4	0.000
		model_ligand	3	0.000	0.333	0.000	1.424	0.234	3	0.064
			4	0.000	0.000	0.000	0.000	0.007	4	0.000
PKM2	3ME3	Apo2ph4	3	0.004	0.185	0.008	1.768	0.105	6	0.044
		best_ligand	3	0.041	0.005	0.010	18.508	0.000	4	0.032
		model_cnn_1	3	0.000	0.000	0.000	0.000	0.000	3	0.000
			4	0.000	0.000	0.000	0.000	0.000	4	0.000
		model_cnn_2	3	0.000	0.000	0.000	0.000	0.001	3	0.000
			4	0.000	0.000	0.000	0.000	0.000	4	0.000
		model_cnn_3	3	0.000	0.000	0.000	0.000	0.000	6	0.000
			4	0.000	0.000	0.000	0.000	0.000	6	0.000
		model_cnn_4	3	0.000	0.000	0.000	0.000	0.000	3	0.000
			4	0.000	0.000	0.000	0.000	0.000	4	0.000
		model_cnn_5	3	0.002	0.035	0.004	0.951	0.037	3	0.010
			4	0.000	0.000	0.000	0.000	0.000	4	0.000
		model_ligand	3	0.005	0.059	0.008	2.059	0.028	3	0.018
			4	0.006	0.013	0.008	2.608	0.005	4	0.008
PPARG	5Y2T	Apo2ph4	3	0.009	0.556	0.019	1.809	0.307	4	0.101
		best_ligand	3	0.056	0.148	0.081	10.671	0.014	3	0.078
		model_cnn_1	3	0.006	0.333	0.012	1.128	0.295	3	0.062
			4	0.000	0.000	0.000	0.000	0.000	5	0.000
		model_cnn_2	3	0.000	0.000	0.000	0.000	0.023	3	0.000
			4	0.000	0.000	0.000	0.000	0.000	5	0.000
		model_cnn_3	3	0.000	0.000	0.000	0.000	0.000	3	0.000
			4	0.000	0.000	0.000	0.000	0.000	5	0.000
		model_cnn_4	3	0.000	0.000	0.000	0.000	0.018	3	0.000
			4	0.000	0.000	0.000	0.000	0.000	9	0.000
		model_cnn_5	3	0.000	0.000	0.000	0.000	0.002	3	0.000
			4	0.000	0.000	0.000	0.000	0.000	4	0.000
		model_ligand	3	0.030	0.037	0.033	5.820	0.006	3	0.032
			4	0.000	0.000	0.000	0.000	0.000	4	0.000
PPARG	5Z5S	Apo2ph4	3	0.016	0.111	0.028	3.049	0.036	5	0.038
		best_ligand	3	0.500	0.111	0.182	96.037	0.001	4	0.403
		model_cnn_1	3	0.000	0.000	0.000	0.000	0.016	3	0.000
			4	0.000	0.000	0.000	0.000	0.000	4	0.000
		model_cnn_2	3	0.004	0.111	0.008	0.834	0.133	3	0.027
			4	0.000	0.000	0.000	0.000	0.003	4	0.000
		model_cnn_3	3	0.013	0.074	0.022	2.478	0.030	3	0.027
			4	0.000	0.000	0.000	0.000	0.006	4	0.000
		model_cnn_4	3	0.009	0.148	0.017	1.727	0.086	3	0.040
			4	0.000	0.000	0.000	0.000	0.004	4	0.000
		model_cnn_5	3	0.008	0.704	0.017	1.621	0.434	3	0.103
			4	0.000	0.000	0.000	0.000	0.036	4	0.000
		model_ligand	3	0.007	0.111	0.013	1.372	0.081	3	0.030
			4	0.000	0.000	0.000	0.000	0.007	4	0.000

Target	system	model	Min_Feats	precision	recall	f1	enrichment	hit_rate	Num_Feats	Guner-Henry
TP53	4AGQ	Apo2ph4	3	0.009	0.139	0.017	0.469	0.297	4	0.029
		best_ligand	3	0.058	0.177	0.087	3.069	0.058	3	0.083
		model_cnn_1	3	0.011	0.013	0.012	0.612	0.021	3	0.012
			4	0.000	0.000	0.000	0.000	0.000	4	0.000
		model_cnn_2	3	0.013	0.013	0.013	0.674	0.019	3	0.012
			4	0.000	0.000	0.000	0.000	0.000	4	0.000
		model_cnn_3	3	0.000	0.000	0.000	0.000	0.001	3	0.000
			4	0.000	0.000	0.000	0.000	0.000	4	0.000
		model_cnn_4	3	0.014	0.076	0.023	0.738	0.103	3	0.026
			4	0.000	0.000	0.000	0.000	0.000	5	0.000
		model_cnn_5	3	0.000	0.000	0.000	0.000	0.000	4	0.000
			4	0.000	0.000	0.000	0.000	0.000	4	0.000
		model_ligand	3	0.014	0.025	0.018	0.766	0.033	4	0.017
			4	0.014	0.025	0.018	0.766	0.033	4	0.017
VDR	3A2I	Apo2ph4	3	0.002	0.180	0.004	0.786	0.229	5	0.036
		best_ligand	3	0.010	0.014	0.012	4.172	0.003	5	0.011
		model_cnn_1	3	0.002	0.335	0.004	0.825	0.406	3	0.051
			4	0.002	0.045	0.004	0.802	0.056	4	0.012
		model_cnn_2	3	0.000	0.000	0.000	0.000	0.000	3	0.000
			4	0.000	0.000	0.000	0.000	0.000	4	0.000
		model_cnn_3	3	0.026	0.001	0.002	10.594	0.000	4	0.020
			4	0.026	0.001	0.002	10.594	0.000	4	0.020
		model_cnn_4	3	0.002	0.334	0.005	0.979	0.341	3	0.056
			4	0.000	0.000	0.000	0.000	0.000	8	0.000
		model_cnn_5	3	0.002	0.247	0.005	0.927	0.266	3	0.047
			4	0.002	0.038	0.004	0.760	0.051	4	0.010
		model_ligand	3	0.003	0.094	0.005	1.056	0.089	3	0.023
			4	0.000	0.000	0.000	0.000	0.000	5	0.000
